# Senescence in neurons: an open issue

**DOI:** 10.18632/aging.203329

**Published:** 2021-07-14

**Authors:** Jordi Bruna, Aina Calls, Victor J. Yuste

**Affiliations:** 1 Neuro-Oncology Unit, Hospital Universitari de Bellvitge-ICO L'Hospitalet (IDIBELL), Barcelona, Spain; 2 Department of Cell Biology, Physiology and Immunology, Universitat Autònoma de Barcelona, Barcelona, Spain; 3 Department of Biochemistry, Institute of Neuroscience, Autonomous University of Barcelona, Bellaterra, Spain

**Keywords:** neuron, senescence, cisplatin, neurotoxicity, chemotherapy-induced peripheral neuropathy

Senescence and aging are two very close linked concepts, not always with clear boundaries, a situation that often induces an undifferentiated use of these terms and misinterpretations regarding their intercalate roles. Outside of teleonomic hypothesis, aging can be accepted as the process of functional decline of cells and organs over time, mechanistically induced by the progressive convergence of damage onto DNA (nuclear and mitochondrial), inefficiently counterbalanced by DNA repair mechanisms [1]. In contrast, senescence (or more accurate, aging-related senescence) is a stress cellular response that constricts its onset to a more limited time window. This cellular state was formerly characterized by the stable cell cycle arrest despite mitogenic stimulus, and resistance to apoptosis, but now it is also recognized that it comes along with heterogeneous morphological and functional changes, and secretion of pro-inflammatory mediators that can spread this response to surrounding cells (paracrine senescence) [2].

The ambiguity in the use of both concepts is furnished by the nonscientific language meaning, and because both processes share triggers (i.e., DNA damage, telomeres shortening, epigenetic changes, and mitochondrial dysfunction), show persistent activation of DNA Damage Response pathways, proteostatic stress, and chronic low-grade inflammation, to finally end in a loss-of-normal cellular or organ function. Moreover, the heterogeneity in the transcriptomic profile in senescence cells and the lack of specific markers, which depend on the inducer, the elapsed time until cell evaluation, and the cell type [3], along with the controversial role about the accumulation of senescent cells in aging tissues as causality or consequence factor, set problems to stablish a clear terminology use, and even arise doubts upon if they really are two different processes or, on the contrary, they are interconnected behaviors.

Conceivably, cells cumulate DNA alterations and sustained activation of repair pathways throughout life (aging), until they achieve a ‘critical damage tolerance’ that can turn out in three potential outcomes. The reactivation of programs enabled during embryogenesis and morphogenesis (the apoptosis’ triggering or alternatively, the entry into senescence), and the neoplastic transformation that in turn, can be stimulated by a senescent microenvironment. These outcomes (senescence and tumorigenesis) are reinforced by the loss of efficiency of the immune system, also affected by the aging process, in the clearance of damaged cells. The preferential exit route adopted by a cell in front of critical damage is currently unknown or, at least, difficult to predict. Probably, it might depend, or be influenced, by the intensity of the damage (extension and velocity of instauration), by the cell cycle status of the affected cells (quiescent, proliferative or differentiated), by the effectiveness of DNA repair mechanisms and antioxidant neutralization systems, and by the regenerative capacity of the affected tissue.

So, an improvement of definitions is needed, either through the identification of differential molecular and/or metabolic signatures for each process, or by the delimitation of a ‘critical damage tolerance’ concept. Until this is achieved, it would be more clarifying to restrict the use of ´senescence´ for cellular processes and aging for tissues and systems. Alternatively, it could be also useful to apply a time-functional perspective to the use of these terms. Loss of normal function in a ‘short period of time’ in response to certain inducers, for senescence; and progressive acquisition of the typical phenotypic response (persistent DNA repair pathways, proteostatic stress, and inflammation) without critical loss of function, for aging.

Recently, the International Cell Senescence Association defined and discussed the key molecular and cellular features of senescence [2]. Despite they acknowledged that post-mitotic cells can acquire senescence-like phenotypes, their recommendations are mainly focused in dividing cells, and maintain the cell-cycle arrest as one of the defining characteristics of the senescence phenotype. This feature does not fit post-mitotic differentiated cells, such as neurons. In any case, at the beginning of the past decade, Diana Jurk and cols. identified other recognized senescence markers in neurons of aged mice [4]. In the same way, we recently demonstrated that the DNA damage induced by platinum drugs in the cisplatin-induced peripheral neuropathy does not induce apoptosis but a senescence-like response in the dorsal root ganglia neurons. This noxious stimulus upregulates the *Cdkn1a* gene and its protein product, increase the expression of senescence-associated β-galactosidase, phospho-H2AX, nuclear factor-kappa B (NF-kB)–p65 proteins, and causes cellular morphological changes, all of them typically recognized as senescence hallmarks [5]. However, if senescence in proliferative cells is only a set of pleiotropic cellular outcomes,-defined by general state categories (such as cell-cycle arrest, the secretion status, macromolecular damage, energy metabolic dysregulation, and certain epigenetic changes) without specific markers, how can we fit similar stress responses in neurons? What are the frontiers to differentiate such stress response from those adaptive physiological cellular responses? Is it necessary to identify signs of previous aberrant activation of neuronal cell cycle, as other authors report in neurodegenerative diseases [6], to recognize a senescence response in neurons? Certainly, it is important to clarify the meaning of cellular senescence, not only for the progress in the understanding of age-related and neurodegenerative diseases, but also for prevalent neurological complications of cancer treatment, like the peripheral neuropathies and probably, the chemobrain, particularly, when senolytic and senomorphic treatments are emerging. In addition, the full comprehension of post-mitotic senescence-like response can provide new clues about how other phenotypically senescent cells can exceptionally re-enter in the cell-cycle, such as the case of some malignant tumor cells,-acquiring in this evolution, drug resistance mechanisms that raise their aggressive behavior [7].

## References

[1] Schumacher B, et al. Nature. 2021; 592:695–703. 10.1038/s41586-021-03307-7 33911272 PMC9844150

[2] Gorgoulis V, et al. Cell. 2019; 179:813–27. 10.1016/j.cell.2019.10.005 31675495

[3] Hernandez-Segura A, et al. Curr Biol. 2017; 27:2652–2660.e4. 10.1016/j.cub.2017.07.033 28844647 PMC5788810

[4] Jurk D, et al. Aging Cell. 2012; 11:996–1004. 10.1111/j.1474-9726.2012.00870.x 22882466 PMC3533793

[5] Calls A, et al. Neuro-oncol. 2021; 23:88–99. 10.1093/neuonc/noaa151 32597980 PMC7850121

[6] Chauhan M, et al. Cell Death Dis. 2020; 11:441. 10.1038/s41419-020-2647-1 32513985 PMC7280246

[7] Saleh T, et al. Cancer Res. 2019; 79:1044–46. 10.1158/0008-5472.CAN-18-3437 30803994

